# Current Features of Aortic Graft and Endograft Infections: A Single-Centre Study of 37 Patients on the Effects of Medical and Surgical Treatment

**DOI:** 10.3390/jcm15135019

**Published:** 2026-06-27

**Authors:** Nathalie Scarpulla, Fabian Patauner, Lorenzo Bertolino, Roberto Andini, Daniela Pinto, Bartolomeo Di Benedetto, Marisa De Feo, Rosa Zampino, Emanuele Durante-Mangoni

**Affiliations:** 1 Department of Advanced Medical and Surgical Sciences, University of Campania ‘Luigi Vanvitelli’, 80138 Naples, Italy; nathalie.scarpulla@studenti.unicampania.it (N.S.); rosa.zampino@unicampania.it (R.Z.); 2 Department of Precision Medicine, University of Campania “Luigi Vanvitelli”, 80138 Naples, Italy; fabian.patauner@unicampania.it (F.P.); lorenzo.bertolino@unicampania.it (L.B.); 3 Unit of Internal Medicine and Transplants, Monaldi Hospital, Piazzale Ettore Ruggieri, 80131 Naples, Italy; roberto.andini@ospedalideicolli.it (R.A.); daniela.pinto@ospedalideicolli.it (D.P.); 4 Unit of Vascular Surgery, Monaldi Hospital, Piazzale Ettore Ruggieri, 80131 Naples, Italy; bartolomeo.dibenedet@ospedalideicolli.it; 5 Department of Translational Medical Sciences, University of Campania “Luigi Vanvitelli”, 80138 Naples, Italy; marisa.defeo@unicampania.it; 6 Unit of Cardiac Surgery, University of Campania “Luigi Vanvitelli”—Monaldi Hospital, 80138 Naples, Italy

**Keywords:** aortic graft and endograft infections, microbiology, therapeutic management, outcomes, aorta

## Abstract

**Objectives:** To assess whether outcomes differ in patients with aortic graft and endograft infections (AGEIs) according to therapeutic approach (medical treatment alone versus combined medical and surgical treatment) and to describe in detail the radiological and microbiological features of these infections. **Methods:** This was a single-centre, observational, retrospective study including patients admitted to Monaldi Hospital, Naples, Italy, with a diagnosis of AGEI between 2005 and 2025. All patients fulfilled MAGIC criteria for definite or suspected AGEI. **Results:** During the study period, 37 patients were enrolled. According to MAGIC criteria, 25 patients had a definite AGEI, while 12 met criteria for suspected infection. A microbiological diagnosis was obtained in 31 patients (84%), mainly from blood cultures (68%). Medical treatment alone was chosen for 19 patients (51%), whereas 18 patients received combined medical and surgical treatment. Crude 30-day, 90-day and 1-year mortality estimates were similar between treatment groups, whereas crude 3-year mortality was numerically higher in patients receiving medical treatment alone. Kaplan–Meier analysis showed a non-significant difference in survival according to treatment strategy (log-rank *p* = 0.160). **Conclusions:** AGEIs remain a severe and often fatal complication. In this small retrospective cohort, no statistically significant survival difference was observed between treatment strategies, although a clinically meaningful benefit of surgery cannot be excluded. Graft location was associated with distinct microbiological patterns and may help guide empirical antimicrobial therapy.

## 1. Introduction

Aortic graft and endograft infections (AGEIs) are uncommon but often devastating conditions, occurring in 1–5% of implant carriers. Incidence is seemingly increasing, likely due to advances in graft design and implantation techniques that translate into raising numbers of patients eligible for surgery [1]. The dreadful nature of this condition is denoted by the high morbidity burden and very high mortality rates, ranging from 10 to 25% and up to 50% at 30 days and 1 year, respectively [2].

Considering the heterogeneity and complexity of AGEIs, careful management of these cases is vital, possibly under the responsibility of a multidisciplinary team in the context of tertiary care centres [3]. The microbiology of AGEIs is constantly changing. There has been a gradual reduction in the incidence of Gram-positive bacteria, particularly *S. aureus*, and a constant increase in Gram-negative bacilli infections [1]. Due to changes in aetiology, microbiological sampling is now mandatory and must be performed prior to antimicrobial therapy initiation [4].

Surgical removal of the infected graft and appropriate, possibly targeted antibiotic therapy represent the mainstays of treatment [5]. However, when removal is not feasible, combined approaches with graft-retaining procedures and antibiotic therapy have proved, in small case series, to be effective in reducing the burden of the infection [6]. Sometimes, due to comorbidities and/or technical hurdles, patients may not be suitable for any surgical approach; in these cases, long-term antibiotic therapy remains the only treatment option [7].

The most appropriate management is oftentimes a tailored approach that takes into consideration patient-related factors, graft-related factors and local complications of the infection [8]. The current published evidence largely derives from systematic reviews based on old and small case series and is also weakened by the lack of randomised controlled trials [9].

To contribute to the increasing evidence in this field of cardiovascular infections, we conducted this study based on our centre’s clinical experience with AGEIs, with the aim to assess whether the outcome of patients differed according to the therapeutic approach (medical treatment alone versus a combined medical and surgical treatment). We also aimed to provide an analytical description of contemporary clinical, radiological and microbiological features of AGEI and to assess whether any of these variables might have an impact on clinical outcomes.

## 2. Materials and Methods

### 2.1. Study Design

This was a single-centre, observational, retrospective study, involving all patients diagnosed with AGEI between January 2005 and January 2025 at the Monaldi Hospital, University of Campania “Luigi Vanvitelli” (AGEIROS). Patients were identified through consultation and in-patient discharge records. Patients fulfilling MAGIC criteria [10] for suspected or definite AGEI were included in the study. We excluded those with suspected or definite native infective aortitis, patients with extra-cavitary (peripheral) arterial graft infections, and patients with composite valve-grafts implanted with Bentall technique.

This study was approved by the Campania 2 Ethics Committee (prot. N. AOC/20606/2025).

### 2.2. Data Collection and Variables of Interest

Cases were identified by searching all discharge records using terms “vascular prosthesis”, “vascular endoprosthesis”, “aortic prosthesis” and “aortic endoprosthesis”. As prosthesis history is routinely documented in the clinical history section of every record, this search captured all patients admitted to our department with an aortic vascular prosthesis. All matching records were then reviewed to identify cases of prosthesis infection. Data was collected after thorough evaluation of clinical records from all patients’ electronic charts available for each hospitalisation or outpatient visit and uploaded on an Excel datasheet. Data collected included past medical history, comorbidities, vascular graft characteristics, symptoms at presentation and therapeutic management (surgical and medical). Congestive heart failure (CHF), chronic obstructive pulmonary disease (COPD), ischaemic heart disease (IHD), diabetes mellitus, liver disease, malignant neoplasia and chronic kidney disease (CKD) were considered as the main co-morbidities. The analysis also included imaging data (all patients underwent either contrast-enhanced computed tomography (CT) or 18F-FDG positron emission tomography/CT [PET-CT] scan or both), haematochemical exams (full blood count, C-reactive protein [CRP], creatinine, ferritin) and microbiological data. Median duration of antibiotic treatment was also collected, as well as all molecules used for both empirical and targeted therapy.

### 2.3. Definitions

For every patient, the time-to-infection, defined as the interval between graft implantation and infection diagnosis, was calculated; when patients had more than one implant, time-to-infection was considered from the most recent prosthesis to infection diagnosis. Whenever the time-to-infection was shorter than 4 months [9], we considered the infection as an ‘early-onset’. Microbiological findings were interpreted according to the MAGIC criteria and considering the clinical radiological context. These criteria account for the distinction between possible contamination and true infection by assigning different diagnostic weight to microbiological results according to the type of sample. Potential contaminants were considered clinically relevant only when isolated from more than one independent sample and when consistent with the overall clinical scenario. Contrast-enhanced CT scans were defined as positive if they met minor or major criteria of the MAGIC classification for the radiological domain [10]. In contrast, PET-CT scans were defined as positive if the nuclear radiologist reported increased and non-homogenous contrast uptake consistent with AGEI. Multi-drug-resistant (MDR) status was determined from the available antimicrobial susceptibility data. In cases where only partial antibiogram results were documented, MDR classification was applied solely when the available information was sufficient; otherwise, MDR status was considered indeterminate.

To account for potential temporal confounders, patients were stratified into two groups according to the median year of diagnosis of the entire cohort. Patients diagnosed before 2014 were classified as the “earlier cohort”, while those diagnosed from 2014 on were classified as the “later cohort”. Surgical approaches were considered and classified as ‘curative’ when complete graft excision was performed and ‘conservative’ in the case of graft preserving approaches.

### 2.4. Statistical Analysis

Continuous variables were presented as median and interquartile range (IQR) and categorical variables as number and percentage. Significance of differences among continuous variables and categorical variables was analysed with Mann–Whitney and chi-square test or Fisher’s exact test, respectively, except for crude mortality rates which, given the limited statistical power, are presented descriptively only. Multivariable analysis was performed using binary logistic regression. Variables with *p* < 0.05 at univariate analysis were entered into the model. Considering the limited sample size, the results are to be considered exploratory. Sensitivity analysis was performed restricting the cohort to definite AGEI cases only. Earlier and later cases were compared as defined above to assess the potential impact of temporal confounders on clinical and microbiological findings and outcomes. Survival analysis was performed drawing Kaplan–Meier curves, with log-rank test to assess differences between subgroups. Missing data were handled by complete-case analysis; no imputation was performed. The number of missing values for each variable is reported in the table footnotes. A *p*-value < 0.05 was defined as statistically significant. Data analysis was carried out with Statistical Package for Social Sciences v. 30 (SPSS Inc., Chicago, IL, USA).

## 3. Results

### 3.1. Demographics and Comorbidities

In the study period (2005–2025), 54 patients received a diagnosis of aortic infection, of whom 37 met inclusion criteria. Included patients were almost all male (92%), with a median age of 71.0 years (IQR 65.0–76.0). Baseline patient profiles are summarised in Table 1.

A very high burden of comorbidities was observed. Twenty-one patients (62%) had a surgical graft and 13 (38%) carried an endovascular graft. In terms of graft location, abdominal grafts, thoracic grafts, and thoraco-abdominal grafts accounted for 65%, 27% and 8% of the study cohort, respectively. Type of vascular graft was unavailable in three patients.

### 3.2. Infection Characteristics

Median time-to-infection was 3 years (IQR 0.3–6.3). In terms of clinical presentation, the most common presenting complaint was fever in 21 (57%) patients, followed by pain to the graft region in 12 (32%) patients, and symptoms related to embolic events/peripheral ischaemia and haemoptysis in 4 patients each (11%).

According to MAGIC criteria, 25 (68%) patients had features of ‘definite’ AGEI, whereas 12 (32%) met criteria for ‘suspected’ infection.

Infection characteristics are outlined in Table 2.

A microbiological diagnosis was obtained in 31 patients (84%), mostly from positive blood cultures in 21 (68%), while 6 (16%) AGEI cases remained with negative cultures. AGEIs were caused by a single causative agent in 19 patients (61%), whilst polymicrobial infections accounted for the remaining 12 (39%) cases. Among monomicrobial infections, Gram-positives were the causative agents in 14 (74%) cases, with *Staphylococcus aureus* being the most common (43%), followed by coagulase negative staphylococci (CoNS) (36%) and *Enterococcus faecalis*, *Streptococcus gallolyticus* and *Corynebacterium striatum*, each accounting for 1 case. Gram-negatives were found in four (21%) monomicrobial infections, with equal prevalence of *Escherichia coli*, *Klebsiella pneumoniae*, *Enterobacter cloacae* and *Salmonella* spp. One monomicrobial infection was due to *Candida* spp. Polymicrobial infections were caused by a wider range of microorganisms, with a median number of three causative agents (IQR 2–4). Overall, anaerobes were detected in four patients, all with abdominal AGEI.

Positive imaging diagnostic criteria were present on contrast-enhanced CT scan in 23 of 34 (68%) patients and in 18 of 22 (82%) patients who underwent PET-CT scan, as detailed below.

### 3.3. Imaging Findings

Thirty-six patients (97%) underwent at least one investigation, including PET-CT scan and/or contrast-enhanced CT scan, with findings compatible with graft infection in 27 patients (79%). Considering contrast-enhanced CT scan, 23 (68%) patients had a positive radiological finding; the most common imaging findings were organised or soft collections around prosthesis, fluid collections around prosthesis, and periprosthetic air, found respectively in 16 (47%), 15 (44%), and 14 (41%) patients. The PET-CT scan was positive for significant aortic uptake in 18 of 22 patients (82%). Median standardised uptake value (SUV max) in positive PET-CT scans was 8.6 (IQR 6.3–11.8).

### 3.4. Microbial Aetiology Across Patient Subgroups

Analysing early onset AGEIs, monomicrobial infections accounted for six out of eight AGEIs (75%); among them, four patients (73%) had an infection related to Gram-positive cocci and two patients (21%) to Gram-negative bacilli. Comparing early-onset and late-onset infections, the prevalence of MDR bacteria was evaluable for 27 patients, and in this subgroup MDR prevalence was 4 of 7 (57%) for early-onset infections and 7 of 20 (35%) in the late-onset infections (*p* = 0.38).

Comparing polymicrobial and monomicrobial infections, abdominal aortic graft involvement (*p* = 0.012), isolation of Gram-negative bacilli (*p* = 0.001) and presence of aorto-cavitary fistula (*p* = 0.019) were more common in patients with polymicrobial infections (see Supplementary Table S1). In an explanatory multivariable model, Gram-negative bacilli isolation was independently associated with polymicrobial infections (OR 22.05, 95% CI: 1.31–371.81; *p* = 0.032), though the wide confidence interval reflects the limited sample size and warrants cautious interpretation of this result. In contrast, monomicrobial infections were more often associated with thoracic aortic graft involvement (*p* = 0.012). No statistically significant differences emerged in terms of time-to-infection, haemato-chemical data, SUVmax at PET-CT scan and median survival.

Comparison of abdominal and thoracic AGEI features is shown in Supplementary Table S2. Abdominal AGEIs were more commonly due to Gram-negative bacilli infections (62% vs. 0% in thoracic AGEI; *p* = 0.003).

### 3.5. Therapeutic Approach

Medical treatment alone was the only treatment strategy for 19 patients (51% of the study group), whereas 18 (49%) patients were treated with a combined medical and surgical treatment. A successful graft removal was obtained in four (22%) patients in the combined medical and surgical treatment group, whereas in seven (40%) cases a conservative surgical approach was chosen. Unfortunately, data regarding type of surgery was unavailable in the remaining seven patients (details are provided in Supplementary Figure S1).

Regarding antibiotics employed, empirical therapy was given to seven (19%) patients, with penicillins/cephalosporins and fluoroquinolones being the most frequently used molecules. As intravenous targeted therapy, the preferred molecules were penicillins/cephalosporins in 13 of 26 patients (50%) and glycopeptides in 8 (31%) patients (Supplementary Figure S2). Intravenous targeted therapy was switched to an oral antibiotic regimen in 16 patients (43%) with a long-term suppressive intent and upon hospital discharge. Antibiotic duration was available for 19 patients; among them, median duration of antibiotic therapy was 27.0 weeks (IQR 11.5–81.0 weeks). The six patients with culture-negative AGEI were treated with empiric therapy for a median time of 28.5 weeks. The molecules used for empirical therapy in the culture-negative AGEIs were based on the presence of risk factors for MDR organisms and local epidemiology.

### 3.6. Follow up and Outcomes

Median length of follow-up from diagnosis was 805.5 days (IQR 113.3–1916.3). Overall, 19 (51%) patients were followed up until death. Median survival time in these 19 patients was 197.4 days (IQR 69.8–1026.7). Twenty-nine patients were followed up for >3 years and, in this subgroup, mortality at 30 days, 90 days, 1 year and 3 years was 6%, 16%, 36% and 62%, respectively (Table 3).

Comparing therapeutic approaches, patients managed with medical treatment alone had a higher CCI than those receiving combined medical and surgical treatment (*p* = 0.006). As follow-up completeness varied across time points, crude mortality rates were calculated among patients with available follow-up data at each specific time point and should be interpreted descriptively. Crude 30-day, 90-day, and 1-year mortality rates were similar between groups. Crude 3-year mortality was calculated among the 29 patients with a follow-up longer than 3 years. In this subgroup, mortality rate was numerically higher among patients receiving medical treatment alone compared to those receiving combined medical and surgical treatment (12/14 [86%] vs. 6/15 [40%]) (Table 4).

Restricting the analysis to definite AGEI cases did not substantially change the results. The pattern was consistent with the main analysis despite the limited sample size (Supplementary Table S3).

**Figure 1:**
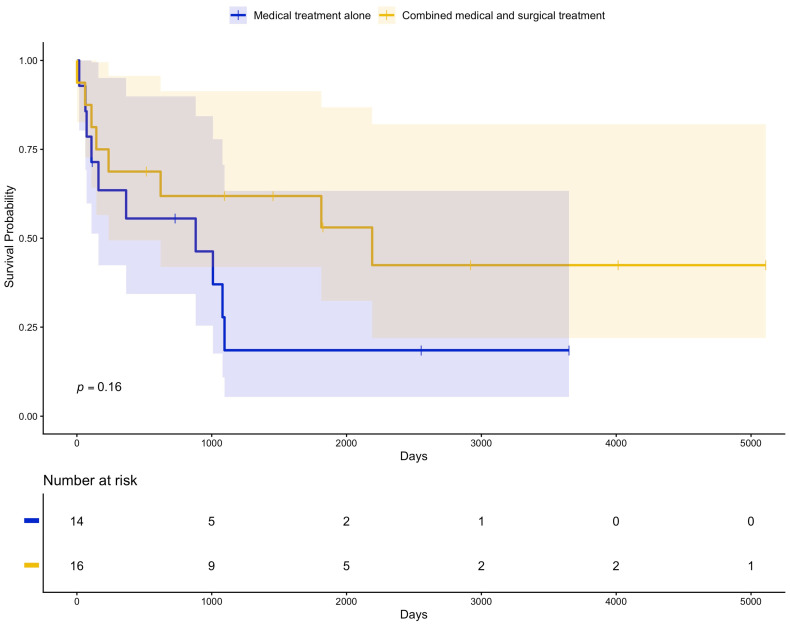
Kaplan–Meier curves showing survival during follow up in combined medical and surgical treatment (yellow line) vs. medical treatment alone (blue line) groups (log-rank test *p* = 0.160).

Comparing Kaplan–Meier curves, a non-significant trend towards a better long-term survival emerged for patients treated with combined medical and surgical treatment (log-rank test *p* = 0.160) (Figure 1).

**Figure 2:**
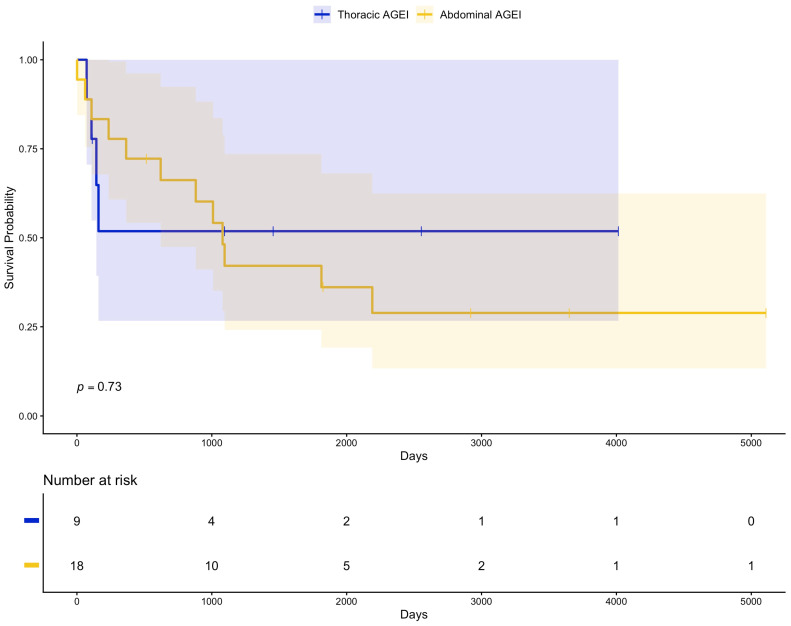
Kaplan–Meier curves showing survival during follow in abdominal AGEIs (yellow line) and thoracic AGEIs (blue line) groups (log-rank test *p* = 0.729).

No difference in survival was found according to AGEIs’ location (log-rank test *p* = 0.728) (Figure 2).

**Figure 3:**
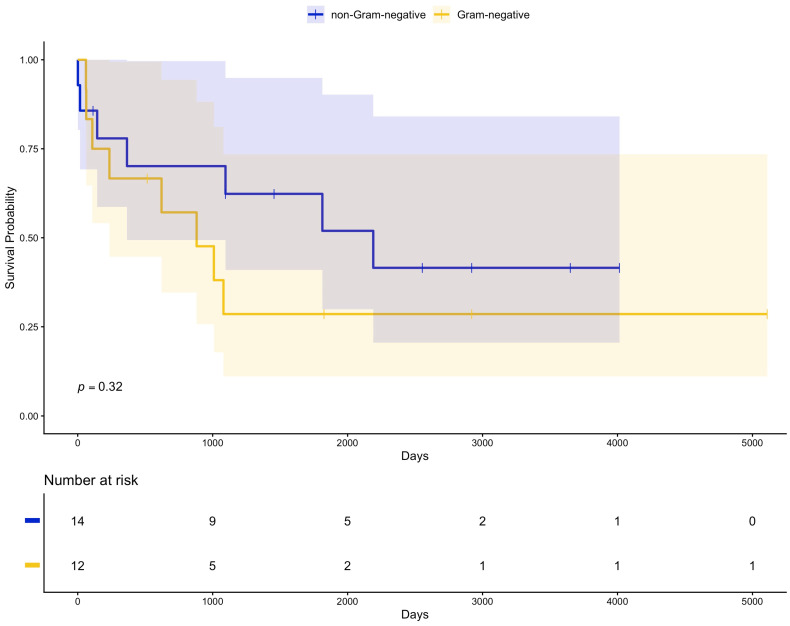
Kaplan–Meier curves showing survival during follow-up in Gram-negative AGEIs (yellow line) and non-Gram-negative AGEIs groups (blue line) (log-rank test *p* = 0.316).

Conversely, a trend for shorter survival was observed for infections due to Gram-negative bacilli compared to non-Gram-negative microorganisms (log-rank test *p* = 0.316) (Figure 3).

To evaluate the potential impact of evolving clinical practice on our clinical results, we performed a stratification by year of treatment (earlier cohort and later cohort); while endograft implantation was more frequent in the later period (*p* < 0.01), reflecting broad trends in vascular surgery, non-significant differences were observed in AGEI treatment strategies or related mortality (Supplementary Table S4).

## 4. Discussion

In this study, we retrospectively analysed 37 contemporary cases of AGEI, highlighting multiple aspects relevant to clinical management of these infections: a) patients managed with a combined medical and surgical treatment showed a non-significant trend towards a better survival and b) microbiological patterns differed according to graft location, which may help in the choice of empirical therapy. To the best of our knowledge, this is one of the few studies including exclusively graft and endograft infections of thoracic and abdominal aorta where therapeutic approaches are compared.

In terms of management, guidelines recommend surgical graft removal in AGEIs [9]. Graft removal surgeries are complicated by high technical hurdles and high perioperative mortality [11], such that not all patients are fit for surgery. The current literature shows contrasting evidence, with some studies favouring surgery for survival [12,13] and others supporting conservative approaches in selected contexts [14,15].

Our study demonstrated a lower 30-day mortality rate compared with two large meta-analyses available in the literature [16,17]. Higher CCI among the medical treatment alone group suggested that these patients were more likely to be deemed unfit for surgery compared to those who underwent surgery. Notably, crude 30-day, 90-day and 1-year mortality were similar between groups, with higher mortality in the medical treatment alone group evident at 3 years. Similarly, Kaplan–Meier survival analysis showed similar rates of mortality in the short and medium term, but a tendency toward improved survival in the long-term for patients receiving combined medical and surgical treatment. Systematic reviews by Moulakakis et al. and Kahlberg et al., despite failing to demonstrate a clear-cut benefit of surgery, also observed a trend for a better outcome of patients treated with surgery, supporting this approach when technically feasible [13,18]. Our results were consistent with these findings, suggesting a possible but statistically not significant benefit of surgery when feasible.

Our analysis showed that abdominal AGEIs are more frequently polymicrobial and more often associated with Gram-negative bacilli, in line with previous studies [19]. Conversely, thoracic AGEIs are often monomicrobial and associated with Gram-positive bacteria. In line with the previous literature, our findings suggest that graft location should be considered among the factors guiding empirical antimicrobial therapy [20]. An approach based on the same empirical therapy suggested for prosthetic left sided infective endocarditis could be employed for thoracic AGEIs. On the other hand, a wider spectrum, covering Gram-negative bacteria, should be considered when treating abdominal AGEIs [21,22].

The understanding of microbiological patterns is crucial to start appropriate empirical therapy, and this could be fundamental for patient survival as demonstrated by Sixt et al. [23].

Given the strong association between aortic fistula and polymicrobial infections, when facing this aetiology, clinicians must take all efforts to find and localise aorto-visceral fistulas. Moreover, the association between Gram-negative and polymicrobial infections should be carefully considered in the therapeutic decision making.

Our study has several limitations, mainly related to the number of patients included in the study that likely impacted its statistical power; however, the small sample size reflects the rarity of AGEIs rather than a recruitment failure. This limitation may have prevented detecting a difference in mortality in relation to therapeutic approaches and precluded multivariable analysis and propensity score analyses for the comparison between treatment strategy and outcomes, leaving this comparison exposed to indication bias. We also lacked standardised disease severity scores, which constrained confounder identification in the mortality analysis. Furthermore, detailed resistance phenotypes were not consistently documented in clinical charts, implying that concordance between empirical therapy and susceptibility testing could not be reliably assessed. Another limitation was related to the inclusion of both definite and suspected AGEI cases according to MAGIC criteria, which introduced some degree of diagnostic heterogeneity. Nevertheless, when the analysis was repeated on definite cases only, the results were consistent with the full cohort, so the inclusion of suspected cases did not appear to have substantially influenced the descriptive findings. Despite extreme efforts made to ensure data accuracy, the retrospective nature of the analysis increased the risk of detection and under-reporting bias. Furthermore, it is worth considering that patients who underwent any kind of surgery were all considered in the combined medical and surgical treatment group may have introduced heterogeneity, potentially obscuring the true benefit of complete graft explantation. This study was conducted in a tertiary care facility and referral centre for cardiovascular infections, and so was also subject to referral bias.

## 5. Conclusions

AGEIs remain a potentially fatal complication. Graft localisation influences microbiological patterns and should be considered before starting empirical therapy. Optimal management should prioritise microbiological diagnosis to convey appropriate antibiotic therapy. In this small retrospective cohort, no statistically significant survival difference was observed between medical treatment alone group and combined medical and surgical treatment group; however, given the limited sample size, a clinically meaningful effect of surgery cannot be excluded. Our findings are therefore hypothesis-generating and require confirmation in adequately powered prospective multi-centre studies.

## Figures and Tables

**Table 1:** Baseline characteristics of the 37 aortic graft and endograft infection cases.

Total number	37
Age, years, median [IQR]	71.0 [65.0–76.0]
Male gender, n (%)	34 (92)
Female gender, n (%)	3 (8)
Comorbidities	
Charlson comorbidity index, median [IQR]	4.0 [3.0–6.0]
History of smoking, n (%)	18 (49)
Chronic obstructive pulmonary disease, n (%)	22 (60)
Diabetes mellitus, n (%)	6 (16)
Malignant neoplasia, n (%)	7 (19)
Chronic kidney disease, n (%)	14 (38)
Ischaemic heart disease, n (%)	16 (43)
Congestive heart failure, n (%)	1 (3)
Peripheral vascular disease, n (%)	37 (100)
Cerebrovascular disease, n (%)	6 (16)
Renal replacement therapy (intermittent haemodialysis), n (%)	0 (0)
Immunodeficiencies, n (%)	0 (0)
Statin therapy, n (%)	17 (46)
Graft characteristics	
Surgical graft, n (%) ^a^	21 (62)
Endograft, n (%) ^a^	13 (38)
Thoracic, n (%)	10 (27)
Abdominal, n (%)	24 (65)
Thoraco-abdominal, n (%)	3 (8)

^a^ Type of vascular graft was unavailable in 3 patients.

**Table 2:** Infection characteristics of the 37 aortic graft and endograft infection cases.

Total number	37
Time-to-infection, years, median [IQR] ^a^	3.0 [0.3–6.3]
Late onset infection, n (%) ^a^	27 (75)
Clinical features	
Fever, n (%)	21 (57)
Pain in the relevant graft region, n (%)	12 (32)
Embolic events, n (%)	4 (11)
Haemoptysis, n (%)	4 (11)
Surgical site swelling, n (%)	3 (8)
Purulent secretions, n (%)	3 (8)
MAGIC criteria fulfilment	
Definite infection, n (%)	25 (68)
Suspected infection, n (%)	12 (32)
Biochemical data	
Creatinine, mg/dL, median [IQR]	1.0 [0.9–1.4]
C-reactive protein, mg/dL, median [IQR]	6.9 [3.45–10.4]
White blood cell count, cells/µL, median [IQR]	8700 [7095–12,225]
Ferritin, mcg/L, median [IQR]	287.0 [133.5–667.8]
Microbiological data	
Microbiological diagnosis, n (%)	31 (84)
Blood culture positive, n (%) ^b^	21 (68)
Other cultures, n (%) ^b^	10 (32)
Monomicrobial, n (%) ^b^	19 (61)
Polymicrobial, n (%) ^b^	12 (39)
Gram-positives, n (%) ^b^	25 (81)
Gram-negatives, n (%) ^b^	14 (45)
Anaerobes, n (%) ^b^	4 (13)
Fungi, n (%) ^b^	2 (7)
Negative cultures, n (%)	6 (16)
Imaging data	
Positive contrast-enhanced CT, n (%)	23 (68)
Positive PET-CT, n (%)	18 (82)
SUV max, median [IQR]	8.6 [6.3–11.8]

^a^ Rates calculated among the 36 patients with available date of graft implantation. ^b^ Rates calculated among the 31 patients with microbiological diagnosis.

**Table 3:** Follow up and outcomes of the 37 aortic graft and endograft infection cases.

Number of patients	37
Length of follow up, days, median [IQR]	805.5 [113.3–1916.3]
Survival, days, median [IQR] ^a^	197.4 [69.8–1026.7]
30-day mortality, n (%) ^b^	2 (6)
90-day mortality, n (%) ^c^	5 (16)
1-year mortality, n (%) ^d^	11(37)
3-year mortality, n (%) ^e^	18 (62)

^a^ Rates calculated among patients followed-up until death (n = 19). ^b^ Rates calculated among patients with complete follow-up data at day 30 (n = 31). ^c^ Rates calculated among patients with complete follow-up data at day 90 (n = 31). ^d^ Rates calculated among patients with complete follow-up data at year 1 (n = 30). ^e^ Rates calculated among patients with complete follow-up data at year 3 (n = 29).

**Table 4:** Aortic graft and endograft infections characteristics and outcomes according to therapeutic approach.

Characteristics	Combined Treatment, n = 18	Medical Treatment Alone, n = 19	*p*-Value
Age, years, median [IQR]	71.0 [60.0–75.7]	70.0 [65.0–70.5]	0.79
Male gender, n (%)	15 (83)	19 (100)	0.10
**Comorbidities and graft characteristics**			
Charlson comorbidity index, median [IQR]	4.0 [1.5–4.5]	6.0 [3–8.25]	0.006
Surgical graft, n (%) ^a^	12 (71)	9 (56)	0.48
Endograft, n (%) ^a^	6 (33)	7 (44)	
Thoracic, n (%)	5 (28)	6 (32)	0.48
Abdominal, n (%)	13 (72)	10 (53)	
Thoraco-abdominal, n (%)	0 (0)	3 (16)	
Definite AGEI, n (%)	14 (78)	11 (58)	0.30
**Follow up and outcomes**			
Time-to-infection, years, median [IQR]	3.0 [0.8–9.3]	2.3 [0.2–6.0]	0.45
Length of follow up, days, median [IQR]	1276.0 [166.0–2737.7]	547.5 [99.0–1083.8]	0.19
Survival, days, median [IQR] ^b^	189.4 [72.7–1514.9]	262.0 [69.75–1027.0]	0.95
30-day mortality, n (%) ^c^	1 (6)	1 (7)	
90-day mortality, n (%) ^d^	2 (13)	3 (20)	
1-year mortality, n (%) ^e^	5 (31)	6 (43)	
3-year mortality, n (%) ^f^	6 (40)	12 (86)	

^a^ Type of vascular graft was unavailable in 3 patients. ^b^ Rates calculated among patients followed-up until death (n = 19). ^c^ Rates calculated among patients with complete follow-up data at day 30 (n = 31). ^d^ Rates calculated among patients with complete follow-up data at day 90 (n = 31). ^e^ Rates calculated among patients with complete follow-up data at year 1 (n = 30). ^f^ Rates calculated among patients with complete follow-up data at year 3 (n = 29). AGEI, aortic graft and endograft infections; Combined treatment (combined medical and surgical treatment group).

## Data Availability

The dataset used for this article is available on reasonable request from the corresponding author.

## Supplementary Materials

The following supporting information can be downloaded at https://www.mdpi.com/article/10.3390/jcm15135019/s1: Table S1. Aortic graft and endograft infections characteristics and outcomes according to monomicrobial or polymicrobial aetiology. Table S2. Comparison of abdominal and thoracic aortic graft and endograft infection cases; Table S3. Sensitivity analysis on definite aortic graft and endograft infections characteristics and outcomes according to therapeutic approach; Table S4. Aortic graft and endograft infections characteristics and outcomes according to year of treatment; Figure S1. Surgical approaches in combined medical and surgical treatment group; Figure S2. Antibiotic therapy employed in the 37 aortic graft and endograft infections cases.

## Abbreviations

The following abbreviations are used in this manuscript:

AGEIs/AGEI	Aortic graft and endograft infection(s)
MAGIC	Management of Aortic Graft Infection Collaboration
AGEIROS	Aortic Graft and Endograft Infections—Retrospective Observational Study
CHF	Congestive Heart Failure
COPD	Chronic Obstructive Pulmonary Disease
IHD	Ischaemic Heart Disease
CKD	Chronic Kidney Disease
CT	Computed Tomography
PET-CT	Positron Emission Tomography—Computed Tomography
CRP	C-reactive protein
IQR	Interquartile Range
SPSS	Statistical Package for Social Sciences
CoNS	Coagulase negative staphylococci
SUV max	Standardised uptake value max
OR	Odds Ratio
95% CI	95% Confidence Interval
CCI	Charlson Comorbidity Index
