# Supplementary material for: Current Features of Aortic Graft and Endograft Infections: A Single-Centre Study of 37 Patients on the Effects of Medical and Surgical Treatment
Source: J Clin Med. 2026 Jun 27;15(13):5019. doi: 10.3390/jcm15135019 (PMC13363044; doi:10.3390/jcm15135019)
Supplement: Supplementary file 1 [file jcm-15-05019-s001.zip › jcm-4351082-supplementary.pdf]

Online Supplementary Material to: Current features of aortic graft and endograft infections: a single-centre study of 37 patients on the effects of medical and surgical treatment

Table S1. Aortic graft and endograft infections characteristics and outcomes according to monomicrobial or polymicrobial aetiology

Characteristics	**Monomicrobial, N= 19**	**Polymicrobial, N=12**	P-value
Age, years, median [IQR]	70.0 [60.0-76.5]	72.5 [68.0-76.0]	0.20
Male gender, n (%)	16 (84)	12 (100)	0.26
Comorbidities and graft characteristics			
Charlson comorbidity index, median [IQR]	4.0 [1.5-8.5]	5.0 [4.0-6.0]	0.71
Surgical graft, n (%) [a]	13 (81)	7 (58)	0.24
Endograft, n (%) [a]	3 (19)	5 (42)	0.24
Thoracic, n (%)	8 (42)	0 (0)	**0.012**
Abdominal, n (%)	10 (53)	11 (92)	
Thoraco-abdominal, n (%)	1 (5)	1 (8)	
Microbiological diagnosis			
Gram-positives, n (%)	14 (74)	11 (92)	0.36
Gram-negatives, n (%)	4 (21)	10 (83)	**0.001**
Anaerobes, n (%)	0 (0)	4 (33)	0.12
Fungi, n (%)	1 (5)	1 (8)	1
Biochemical and clinical data			
C-reactive protein, mg/dl, median [IQR]	7.4 [3.4-10.4]	6.65 [2.2-11.2]	0.77
White blood cell count, cells/μl, median [IQR]	8500 [7245-13605]	8055 [6205-8992]	0.53
Aorto-cavity fistula n, (%)	4 (24)	8 (67)	**0.019**
Time-to-infection, years, median [IQR]	2 [0.2-5.4]	6.5 [1.6-10]	0.14
Follow up and outcomes			
Length of follow up, days, median [IQR]	12431 [115-2190]	1080 [234.9-2920]	0.57
Survival, days, median [IQR] [b]	365 [39.0-1051.9]	427.9 [96.8-1263.2]	0.77
30-day mortality, n (%) [c]	2 (13)	0 (0)	
90-day mortality, n (%) [d]	3 (20)	1 (9)	
1-year mortality, n (%) [e]	5 (36)	3 (27)	
3-year mortality, n (%) [f]	8 (57)	5 (50)	

[a] Type of vascular graft was unavailable in 3 patients
[b] Rates calculated among patients with complete follow-up data (n= 15)
[c] Rates calculated among patients with complete follow-up data at day 30 (n= 26)
[d] Rates calculated among patients with complete follow-up data at day 90 (n= 26)
[e] Rates calculated among patients with complete follow-up data at year 1 (n= 25)
[f] Rates calculated among patients with complete follow-up data at year 3 (n= 24)
Analysis was done excluding patients with culture-negative AGEIs

Online Supplementary Material to: Current features of aortic graft and endograft infections: a single-centre study of 37 patients on the effects of medical and surgical treatment

**Table S2. Comparison of abdominal and thoracic aortic graft and endograft infection cases**			
Characteristics	**Abdominal, N= 23**	**Thoracic, N= 11**	P-value
Age, years, median [IQR]	74.0 [69.0-77.0]	70.0 [65.0-70.5]	**0.002**
Male gender, n (%)	21 (91)	12 (100)	1
Comorbidities and graft characteristics			
Charlson comorbidity index, median [IQR]	5.5 [4.0-7.0]	2 [1.0-3.0]	**0.001**
Surgical graft, n (%) [a]	18 (78)	3 (27)	**0.002**
Endograft, n (%) [a]	5 (21)	3 (27)	
Microbiological diagnosis			
Monomicrobial, n (%)	10 (48)	8 (100)	**0.012**
Polymicrobial, n (%)	11 (52)	0 (0)	
Gram-positives, n (%)	16 (70)	8 (100)	0.28
Gram-negatives, n (%)	13 (62)	0 (0)	**0.003**
Anaerobes, n (%)	4 (17)	0 (0)	0.29
Fungi, n (%)	2 (10)	0 (0)	1
Clinical and biochemical data			
Fever at onset, n (%)	12 (67)	8 (80)	0.68
Abdominal/thoracic pain, n (%)	8 (44)	3 (30)	0.68
C-reactive protein, mg/dl, median [IQR]	7.4 [3.4-10.4]	6.55 [5.12-7.10]	0.80
White blood cell count, cells/µl, median [IQR]	8500 [7245-13605]	9385 [7900-13637.5]	0.62
Aorto-cavity fistula, n (%)	8 (40)	3 (28)	0.70
Time-to-infection, years, median [IQR]	4 [0.65- 9.0]	0.8 [0.3-3.0]	0.06
Follow up and outcomes			
Combined treatment, n (%)	13 (57)	5(46)	0.70
Length of follow up, days, median [IQR]	1044.5 [332.5-2372.5]	159.9 [111.5-2005.5]	0.46
Survival, days, median [IQR][b]	751.1 [139.7-1091.3]	126.0 [81.0-155.9]	0.10
30-day mortality, n (%) [c]	1 (5)	0 (0)	
90-day mortality, n (%) [d]	2 (11)	1 (11)	
1-year mortality, n (%) [e]	5 (26)	4 (50)	
3-year mortality, n (%) [f]	11 (61)	4 (50)	

[a] Type of vascular graft was unavailable in 3 patients
[b] Rates calculated among patients with complete follow-up data (n= 16)
[c] Rates calculated among patients with complete follow-up data at day 30 (n= 28)
[d] Rates calculated among patients with complete follow-up data at day 90 (n= 28)
[e] Rates calculated among patients with complete follow-up data at year 1 (n= 27)
[f] Rates calculated among patients with complete follow-up data at year 3 (n= 26)
Analysis was done excluding patients with thoraco-abdominal aortic graft and endograft infections

Online Supplementary Material to: Current features of aortic graft and endograft infections: a single-centre study of 37 patients on the effects of medical and surgical treatment

Table S3. Sensitivity analysis on definite aortic graft and endograft infections characteristics and outcomes according to therapeutic approach

Characteristics	**Combined treatment, n=14**	**Medical treatment alone, n=11**	P-value
Age, years, median [IQR]	72.5.0 [62.0-75.0]	70.0 [65.0-73.0]	0.42
Male gender, n (%)	12 (85)	11 (100)	0.49
Comorbidities and graft characteristics			
Charlson comorbidity index, median [IQR]	4.0 [2-5.0]	6.0 [3-7.25]	0.37
Surgical graft, n (%) [a]	10 (76)	4 (36)	0.10
Endograft, n (%) [a]	3 (23)	7 (63)	
Thoracic, n (%)	5 (21)	6 (55)	0.12
Abdominal, n (%)	11 (79)	5 (45)	0.12
Thoraco-abdominal, n (%)	0 (0)	3 (27)	0.07
Follow up and outcomes			
Time-to-infection, years, median [IQR]	3.6 [2.0-10.0]	4.0 [0.3-6.0]	0.34
Length of follow up, days, median [IQR]	1217.0 [166.7-2737.5]	730.0 [85.0-1087.5]	0.31
Survival, days, median [IQR]b	234.9 [108.0-1812.9]	159.9 [63.0-1080.0]	0.71
30-day mortality, n (%) [c]	1 (8)	1 (10)	
90-day mortality, n (%) [d]	1 (8)	2 (20)	
1-year mortality, n (%) [e]	4 (33)	4 (40)	
3-year mortality, n (%) [f]	5 (45)	9 (90)	

[a] Type of vascular graft was unavailable in 1 patient
[b] Rates calculated among patients with complete follow-up data (n= 14)
[c] Rates calculated among patients with complete follow-up data at day 30 (n= 22)
[d] Rates calculated among patients with complete follow-up data at day 90 (n= 22)
[e] Rates calculated among patients with complete follow-up data at year 1 (n= 22)
[f] Rates calculated among patients with complete follow-up data at year 3 (n= 21)
Combined treatment, combined medical and surgical treatment

Online Supplementary Material to: Current features of aortic graft and endograft infections: a single-centre study of 37 patients on the effects of medical and surgical treatment

Table S4. Aortic graft and endograft infections characteristics and outcomes according to year of treatment

Characteristics	**AGEI earlier cohort, n=18**	**AGEI later cohort, n=19**	P-value
Age, years, median [IQR]	68.0 [62.0-76.0]	72.0 [65.0-76.0]	0.61
Male gender, n (%)	17 (94)	17 (89)	1
Comorbidities and graft characteristics			
Charlson comorbidity index, median [IQR]	6.0 [2-6.5]	4.0 [3-6.25]	0.30
Surgical graft, n (%) [a]	14 (93)	7 (37)	**< 0.01**
Endograft, n (%) [a]	1 (6)	12 (63)	
Thoracic, n (%)	6 (33)	8 (42)	0.95
Abdominal, n (%)	12 (66)	11 (57)	
Thoraco-abdominal, n (%)	0 (0)	3 (15)	
Microbiological and radiological diagnosis			
Gram-positives, n (%)	14 (93)	11 (68)	0.17
Gram-negatives, n (%)	7 (46)	7 (44)	1
Anaerobes, n (%)	3 (20)	1(6)	0.6
Fungi, n (%)	1 (6)	1 (7)	1
Culture negative, n (%)	3 (16)	3 (16)	1
Monomicrobial, n (%)	9 (60)	10 (63)	1
Polymicrobial, n (%)	6 (40)	6 (38)	1
Positive contrast-enhanced CT, n (%)	16 (89)	18 (94)	0.60
Positive PET-CT, n (%)	8 (73)	10 (90)	0.59
Management and outcomes			
Combined treatment, n (%)	8 (44)	10 (53)	0.75
Medical treatment alone, n (%)	10 (56)	9 (47)	
Treatment length, *weeks*, median [IQR]	4.0 [0.3-9.0]	1 [0.2-4.6]	0.30
Time-to-infection, years, median [IQR][b]	3.6 [2.0-10.0]	4.0 [0.3-6.0]	0.34
Length of follow up, days, median [IQR]	1087.0 [117.0-3467.5]	675.5 [102.0-1545.2]	0.27
Survival, days, median [IQR][c]	144.0 [108.0-1080.0]	234.9 [60.9-1008.9]	0.93
30-day mortality, n (%) [d]	0 (0)	2 (11)	
90-day mortality, n (%) [e]	1 (8)	4 (21)	
1-year mortality, n (%) [f]	5 (41)	4 (33)	
3-year mortality, n (%) [g]	7 (58)	11 (64)	

[a] Type of vascular graft was unavailable in 3 patients

[b] Rates calculated among the 36 patients with available date of graft implantation

[c] Rates calculated among patients with complete follow-up data (n= 18)

[d] Rates calculated among patients with complete follow-up data at day 30 (n= 31)

[e] Rates calculated among patients with complete follow-up data at day 90 (n= 31)

[f] Rates calculated among patients with complete follow-up data at year 1 (n= 30)

[g] Rates calculated among patients with complete follow-up data at year 3 (n= 29)

AGEI, aortic graft and endograft infections

Online Supplementary Material to: Current features of aortic graft and endograft infections: a single-centre study of 37 patients on the effects of medical and surgical treatment

Supplementary Figure S1. Surgical approaches in combined medical and surgical treatment group

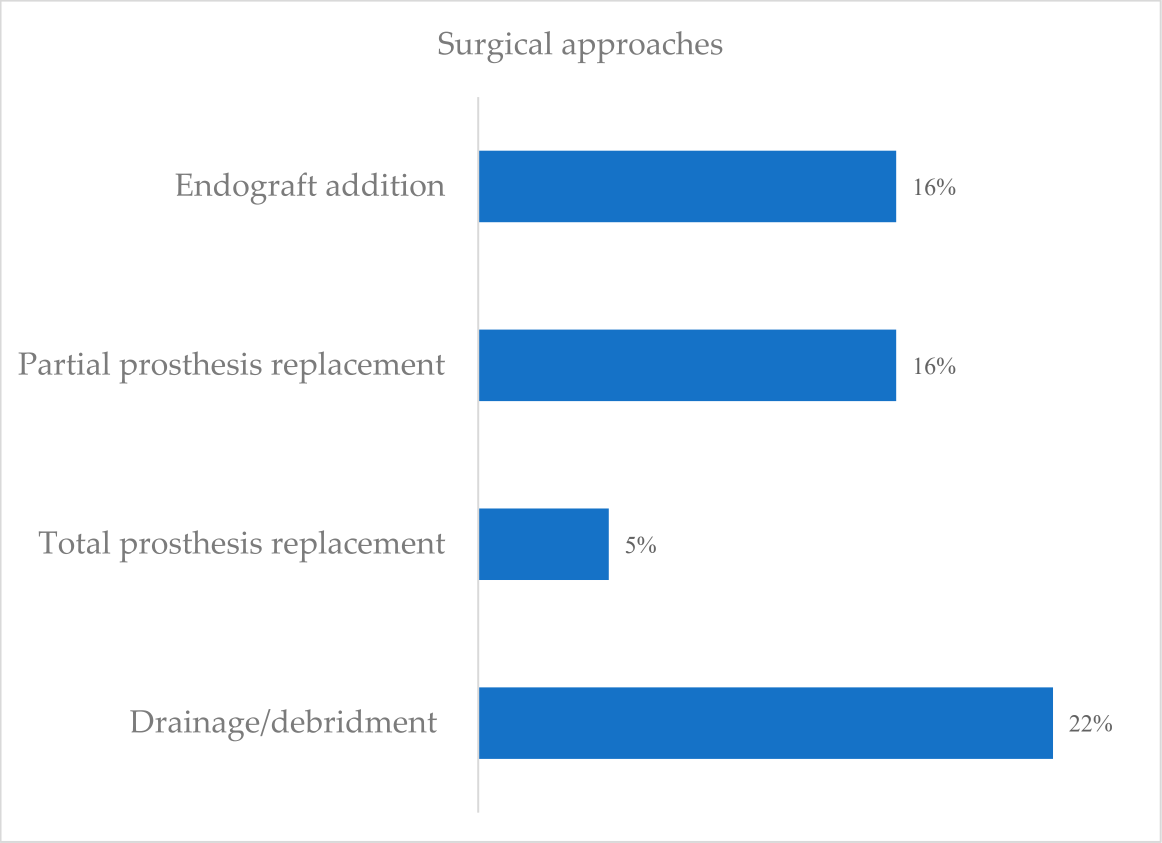

Online Supplementary Material to: Current features of aortic graft and endograft infections: a single-centre study of 37 patients on the effects of medical and surgical treatment

Supplementary Figure S2. Antibiotic therapy employed in the 37 aortic graft and endograft infections cases

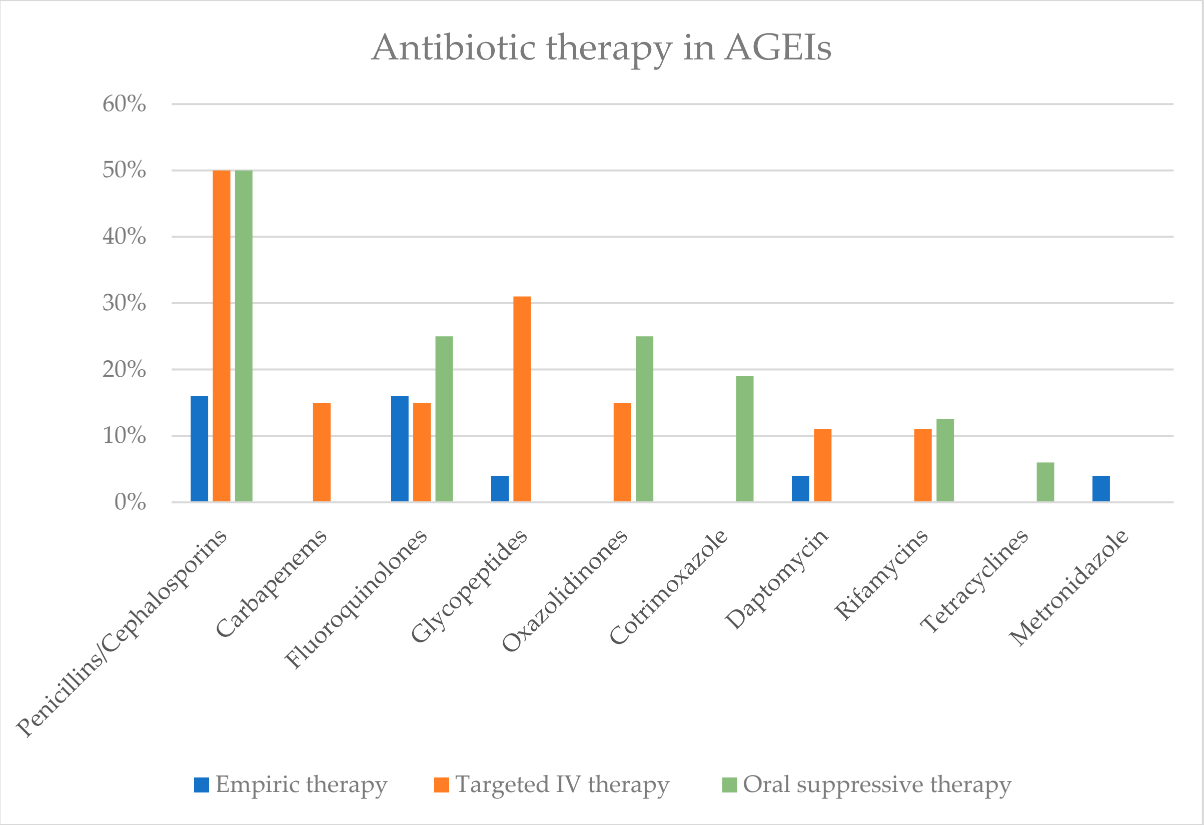

AGEI, aortic graft and endograft infections
